# Synergetic gait prediction and compliant control of SEA-driven knee exoskeleton for gait rehabilitation

**DOI:** 10.3389/fbioe.2024.1358022

**Published:** 2024-01-26

**Authors:** Haojie Liu, Chang Zhu, Zude Zhou, Yunfei Dong, Wei Meng, Quan Liu

**Affiliations:** The School of Information Engineering, Wuhan University of Technology, Wuhan, China

**Keywords:** knee exoskeleton, series elastic actuator, gait prediction, compliant control, personalized trajectory

## Abstract

In recent years, lower limb exoskeletons have achieved satisfactory clinical curative effects in rehabilitating stroke patients. Furthermore, generating individualized trajectories for each patient and avoiding secondary injury in rehabilitation training are important issues. This paper explores the utilization of series elastic actuator (SEA) to deliver compliant force and enhance impact resistance in human-robot interaction, and we present the design of novel knee exoskeleton driven by SEA. Subsequently, the novel gait trajectory prediction method and compliant control method are proposed. The attention-based CNN-LSTM model is established to generate personalized gait trajectories for affected limbs, in which the spatial-temporal attention mechanism is adopted to improve the prediction accuracy. The compliant control strategy is proposed to nonlinearly and adaptively tune impedance parameters based on artificial potential field (APF) method, and active rehabilitation training is carried out in the coordination space to guarantee patient safety. The experimental results based on four healthy subjects demonstrated that synergetic gait prediction model could satisfactorily characterize the coordination movement with higher accuracy. The compliant control could limit the patient’s movement in the safe coordination tunnel while considering personalization and flexibility.

## 1 Introduction

According to the Global Burden of Disease Study, stroke remains the primary cause of the second-highest mortality rate and the third-highest disability rate in the world (Feigin et al., 2022). Patients with lower extremity motor dysfunction after stroke usually show weakened lower extremity muscle strength, limited range of motion, and unstable shift of center of gravity, often accompanied by foot drop and varus deformity (Wang et al., 2017). Knee joint is the most complex joint of human body in structure, which can not only support basic locomotion such as walking, running, and standing, but also effectively dampen the impact force generated during walking. Knee dysfunction caused by neurological diseases is the most common factor leading to gait abnormalities, which severely affects patients’ activities of daily living. Therefore, it is necessary to carry out rehabilitation training and develop the knee exoskeleton to improve mobility (Yan et al., 2022). Research shows that rehabilitation training for patients at the early stage of the stroke has a significantly positive effect (Ballester et al., 2022).

The traditional rehabilitation therapy is time-consuming and laborious, and rehabilitation outcome is limited (Zhang et al., 2021). The lower limb rehabilitation robots can not only reduce the burden of rehabilitation therapists, but also customize the gait trajectory and training intensity (Kim et al., 2019; Cao et al., 2023; Yang et al., 2023). However, traditional knee exoskeletons mostly adopt rigid actuators, which can achieve accurate position control, but lack compliance (Chen et al., 2017). Exoskeletons driven by pneumatic muscles possess high compliance, but how to provide power conveniently is a challenge (Sridar et al., 2018). Series elastic actuator (SEA) intentionally introduces an elastic element between the actuator and output, which has many advantages, including lower reflection coefficient, impact resistance, and more accurate stability control in unconstrained environment (Yu and Lan, 2019). Recently, knee exoskeletons driven by SEA have received increasing attention (Kong et al., 2012; Song et al., 2023). Kong et al. designed the knee exoskeleton with compact rotary series elastic actuator (cRSEA), in which worm gears made no noise and were used to amplify the torque produced by the motor (Kong et al., 2012). Song et al. studied a crank-slider series elastic actuator (CS-SEA), in which crank-slider mechanism could improve the torque effect and the level of transparency, and the experimental results showed the precise force control performance of CS-SEA (Song et al., 2023). However, the weight of the knee exoskeleton can be the burden for patients, and patients vary in body shapes, so the knee exoskeleton should be adjustable to accommodate patients with different physical parameters to improve the adaptability of device and limit the range of motion of the knee exoskeleton to prevent secondary injury.

The predefined gait trajectory is suitable for patients who lack the ability to walk independently in the early stage of rehabilitation. However, in the middle and late stages of rehabilitation, the predefined trajectory may conflict with the patient’s active intention (Zhu et al., 2022; Na et al., 2023). The continuous estimation of human motion intention through gait prediction method exhibits potential for compliant human-robot interaction (Xiong et al., 2021). Gait prediction is mainly based on motion information and physiological information. Motion information mainly includes joint angle, angular acceleration, and plantar pressure (Liu et al., 2017; Mounir Boudali et al., 2019; Sivakumar et al., 2019), and physiological information includes surface electromyography (sEMG) and electroencephalogram (EEG) (Gautam et al., 2020; Morbidoni et al., 2021; Liu et al., 2022). Meanwhile, multi-sensor fusion and multi-feature fusion can realize better prediction accuracy in gait prediction (Mazumder et al., 2016; Arami et al., 2019). Zou et al. proposed a gait prediction model to generate personalized gait trajectory for different subjects, which took the current joint angle of healthy lower limb and the observed historical joint angle of both lower limbs as input, and predicted the future joint angle of the paralyzed leg (Zou et al., 2021).

Human-robot interaction has requirements for control accuracy and safety, but the two criteria are conflict. Compliant control can provide a compromise between control accuracy and safety (Schumacher et al., 2019). Compliant control can control the position and force simultaneously and purposefully, including impedance/admittance control (Kim et al., 2021), hybrid force/position control (Yang et al., 2020), and parallel force/position control (Wang et al., 2011). Compared to hybrid and parallel force/position control, impedance control focuses more on achieving the target relationship between force and position, but does not necessarily track the expected trajectories (Perez-Ibarra et al., 2019; Chen et al., 2020; Sun et al., 2020). Admittance control, also known as position-based impedance control, adjusts the desired trajectory according to force deviation (Almaghout et al., 2022; Huang et al., 2022). Impedance control with adjustable parameters can respond to changes in the external environment (Liu et al., 2020; Wang et al., 2021). Li et al. proposed an iterative learning impedance control method, in which the control objective was the impedance model. This method achieved the desired control accuracy through iteration, which was suitable for the rehabilitation tasks with repeatability (Li et al., 2018). Spyrakos et al. introduced a variable impedance control scheme performing stable trajectory tracking, which ensured the stability of impedance control for flexible-joint robots (Spyrakos-Papastavridis and Dai, 2021).

Currently, AAN algorithm modifies the intervention of the robot according to the patient’s behavior, while adopting virtual walls to guarantee patient safety (Banala et al., 2009; Perez-Ibarra et al., 2019; Asl et al., 2020). Banala et al. developed the force-field controller which applied tangential and normal forces to the ankle, in which the tangential forces moved the ankle along the trajectory, and the normal forces produced virtual tunnel around the desired ankle trajectory (Banala et al., 2009). Asl et al. adopted the force field control term in the velocity field controllers, which acted as the virtual tunnel around the desired trajectory. The forces were applied to the desired trajectory whenever the position of the device deviated more than the safety threshold (Asl et al., 2020). However, the desired trajectory is not individualized for each patient, and the actual trajectory should be modified according to the patient’s motion intentions.

In this paper, the flexible knee exoskeleton driven by SEA is designed, and compliant control scheme is proposed for the rehabilitation of stroke patients. The main contributions of this article can be listed as follows.

1) The ball screw drive system, adjustable design, safety mechanism, dual-purpose interface, and support module are adopted in the knee exoskeleton driven by SEA to improve the safety, compatibility, and utilization rate of the device.
2) The attention-based CNN-LSTM network combined with inter-limb synergy is proposed to generate individualized gait trajectory, in which the spatial-temporal attention mechanism is adopted to improve the prediction accuracy.
3) The compliant control scheme based on artificial potential field (APF) method is proposed to nonlinearly and adaptively modify the impedance parameters according to actual conditions, improving the safety and compliance of individualized gait rehabilitation.

The rest of this paper is organized as follows: Section II demonstrates the detailed information of the knee exoskeleton driven by SEA. Section III introduces the proposed individualized gait trajectory prediction model. Section IV shows the compliant control scheme. Experiments and results are conducted in Section V. Section VI is discussion. Conclusion are presented in Section VII.

## 2 Mechanical design

To enable normal walking, the output torque and angle of the knee exoskeleton in the flexion/extension direction should meet the standards of the human body. The knee angle ranges from −4–66°, and the knee torque ranges from −5–66 *N* ⋅ *m* (Chen et al., 2019). Meanwhile, the exoskeleton should be designed with an adjustable mechanism to adapt to patients with varying physical parameters.

**FIGURE 1:**
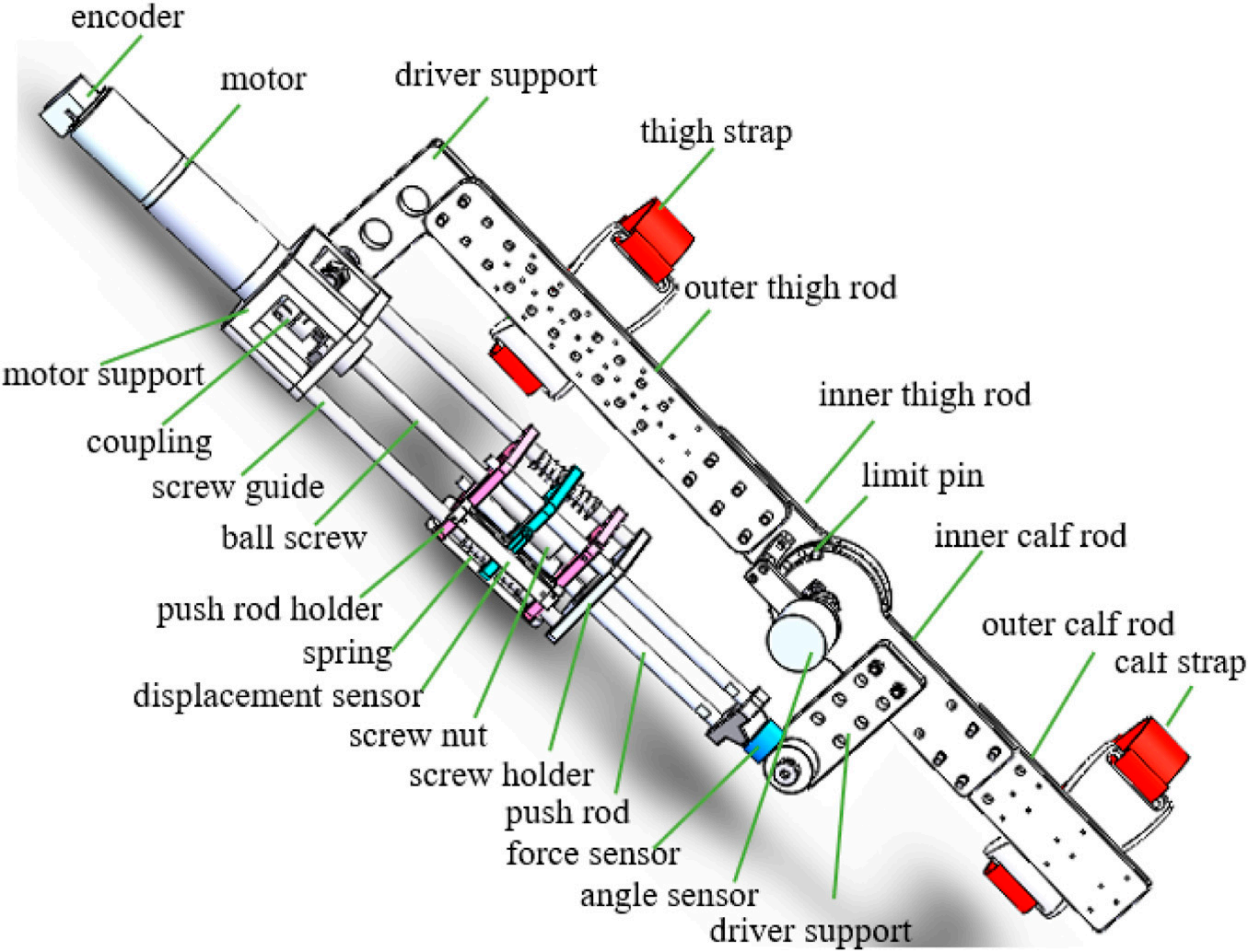
Detailed display of knee exoskeleton driven by SEA (Dong et al., 2022).

The knee exoskeleton driven by SEA designed in this paper is used for unilateral lower limb. The main structure of the knee exoskeleton driven by SEA is shown in Figure 1, which mainly includes six modules, namely, thigh module, calf module, knee module, actuator module, support module, and protection module. The length of the thigh module and the calf module can be adjusted to accommodate patients with different physical parameters, which improves the utilization and adaptability of equipment. The inner calf rod and the drive support can also be adjusted, allowing different force arms to be realized to satisfy different rehabilitation needs.

**FIGURE 2:**
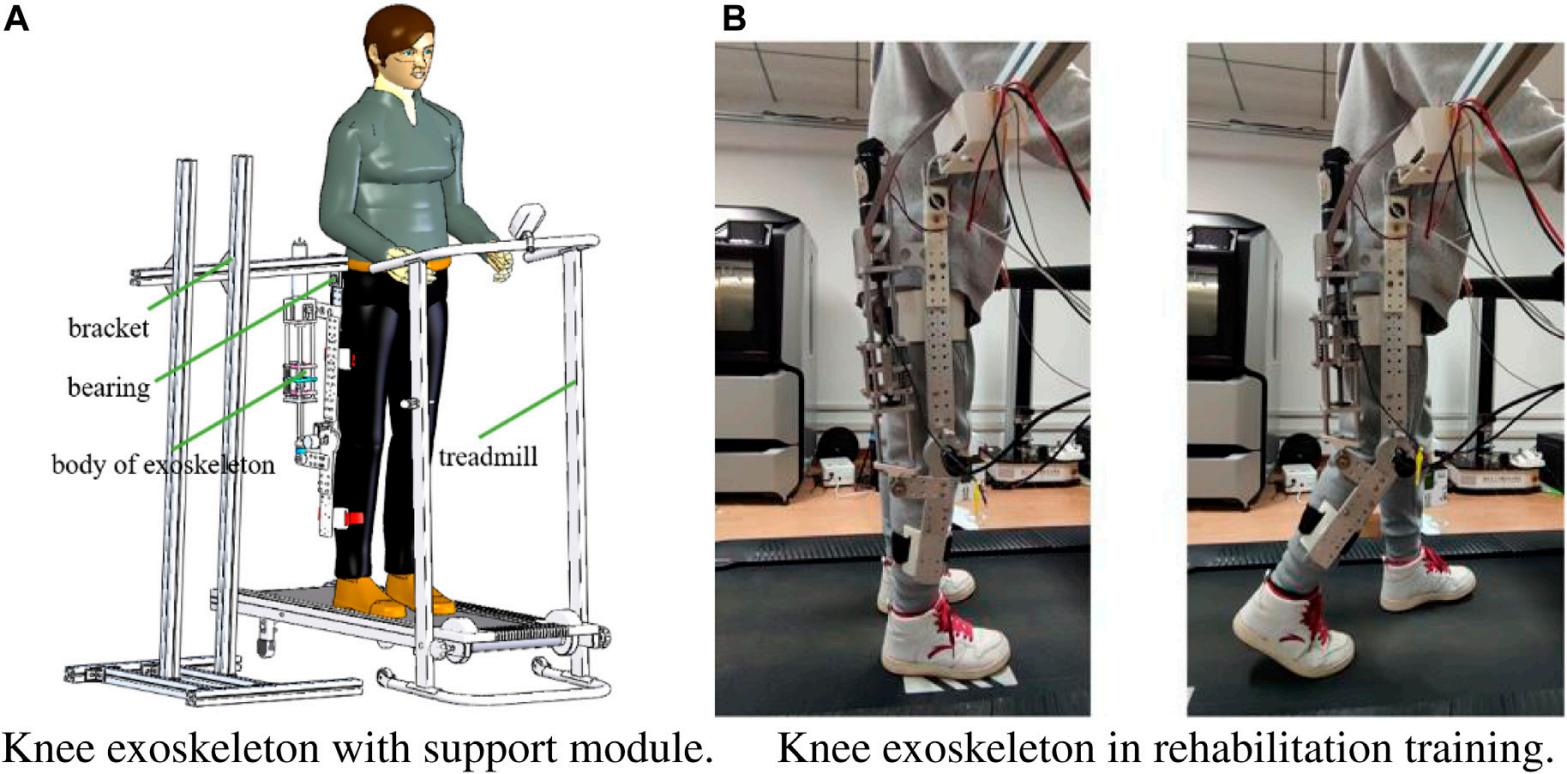
The mechanism of knee exoskeleton. **(A)** Knee exoskeleton with support module. **(B)** Knee exoskeleton in rehabilitation training.

There is a dual-purpose interface at the end of the actuator module, which can realize the normal rotation of the knee joint or calibrate the spring coefficient. The knee module is equipped with safety mechanism to ensure the safety of rehabilitation training. The safety latch can be inserted into the limit hole, and the range of motion of the knee angle can be adjusted by changing the position of the safety latch. The protection module is manufactured through 3D printing technology, and the flexible material enables the protection module to adapt perfectly to the human body. The support module can alleviate the burden on patient, and the patients can wear the knee exoskeleton to achieve gait rehabilitation on the treadmill, as shown in Figure 2A. The support module can also adjust the position of the exoskeleton in three directions. The end of the support module is connected with a bearing, thus promoting unrestricted movement of the hip joint.

The knee exoskeleton adopts SEA as the actuator, as shown in Figure 2B. The SEA is isolated from the load through flexible elements. When the system is impacted, the spring can provide a buffer and absorb energy, which plays a protective role and improve the flexibility of SEA. The angle and displacement sensors are installed at the bottom of the knee module, which can monitor the patient’s motion in real time, providing a hardware basis for compliant and intelligent control. When SEA works, the motor drives the ball screw to rotate, and the nut of the ball screw moves linearly, which compresses or stretches the spring. The force of spring makes the push rod of SEA generate thrust or tension, which can realize the flexion or extension movement of the knee joint. At the same time, the actuator module rotates relatively with the thigh and calf driver support module in a small range. The displacement sensor records the deformation of the spring, and the angle sensor records the flexion and extension angle of the knee joint, and the two signal feeds it back to the control system.

## 3 Individualized gait prediction model

### 3.1 TASK design and data acquisition

Human motor coordination refers to the ability of the neurobiological motor system to generate complex movements involving multiple limbs or joints. Various types of coordinated movements can be executed by the lower limb, including sitting/standing, squatting/jumping. The most common coordinated movement is walking, which is a fundamental athletic skill for other activities.

In this paper, a variety of lower limb coordinated movement tasks using the knee joint are designed. The subjects walked on treadmills at different speeds and slopes. The speeds included 0.5 km/h, 1.5 km/h, and 3.0 km/h, and the slopes included 0°, 4°, and 8°. The slope 0° represent that the human walks on flat ground. The subjects initiated a gait cycle with the right heel touching the ground and end the gait cycle with the next right heel touching the ground. The gait trajectory is the knee joint position trajectory during walking in this paper (Tanghe et al., 2020; Challa et al., 2022; Song et al., 2023).

**FIGURE 3:**
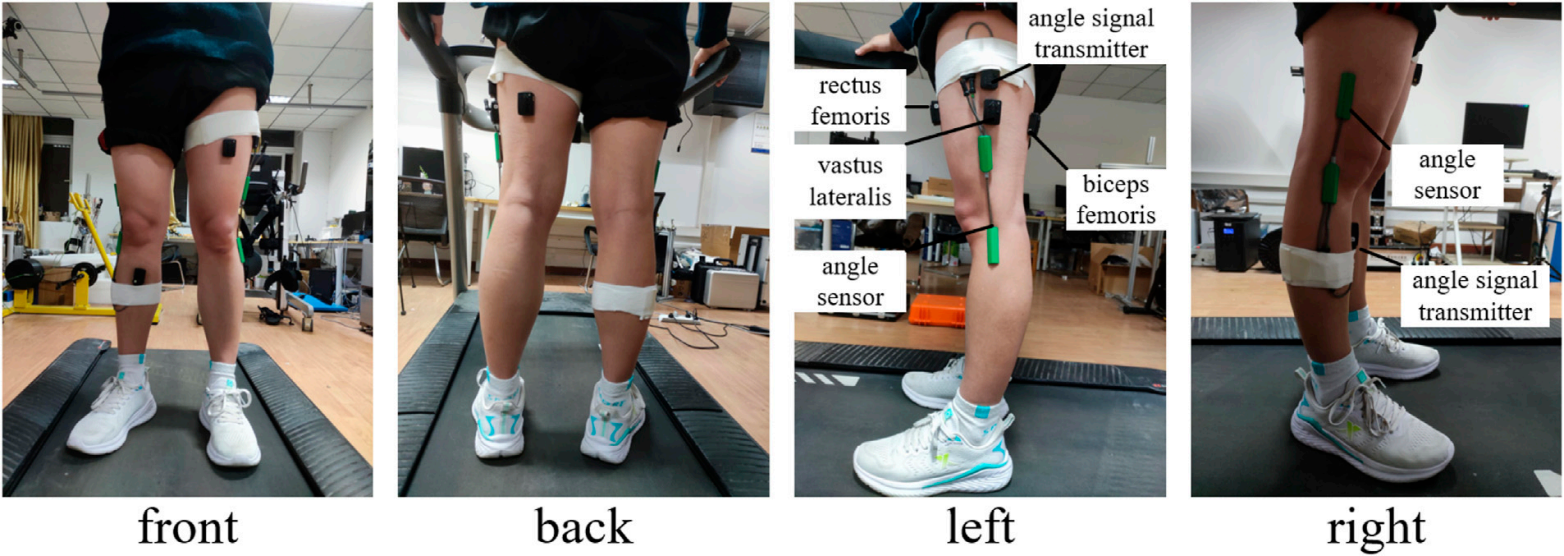
Position of the sEMG and angle sensor.

The coordinated movement data of the subjects are collected and recorded by the Delsys sEMG signal acquisition system. The position of the sEMG and angle sensors is displayed in Figure 3. The sEMG sensors are attached with special double-sided adhesive tape to the three muscles closely associated with the movement of knee joint, namely, rectus femoris, vastus lateralis, and biceps femoris. Two angle sensors are attached to the knee joints with ordinary double-sided tape.

Four healthy subjects were invited to participate in the data collection. This trial has been approved by Human Participants Ethics Committee from Wuhan University of Technology, and written informed consent was obtained from each participant. Participants walked while imitating patients with left lower limb injuries, with a reduction in force produced by the left lower limb muscles and an increase in force mainly produced by the right lower limb muscles. The data collected in the experiment were the sEMG of the three muscles and knee angle signal of the healthy lower limb, as well as knee angle signal of the affected lower limb.

### 3.2 Attention-based CNN-LSTM model

Synergy mechanism is adopted in statistical regression to extract couplings between limbs in healthy synergetic motion. The synergetic gait prediction model can generate the individualized gait trajectory of the affected lower limb based on the sEMG signal and the knee joint angle of the healthy lower limb. Convolutional neural network (CNN) and long short-term memory (LSTM) neural network are widely applied in gait prediction. Standard CNN model is well suited for handling spatially autocorrelated data, which is unsuitable for dealing with complex and long-term dependencies. In contrast, LSTM model is more suitable in handling temporal autocorrelated data. Therefore, the hybrid CNN-LSTM model can effectively improve forecasting performance. Furthermore, the attention model can assign weights to important features, thus enhancing the prediction accuracy (Thakur and Biswas, 2022; Xu et al., 2022). Individualized gait prediction typically involves the collection and analysis of the data specific to an individual, such as motion capture data, the ground reaction forces, and sEMG data. Machine learning algorithms are adopted to analyze the data and develop personalized models that can accurately predict the individual’s gait characteristics.

**FIGURE 4:**
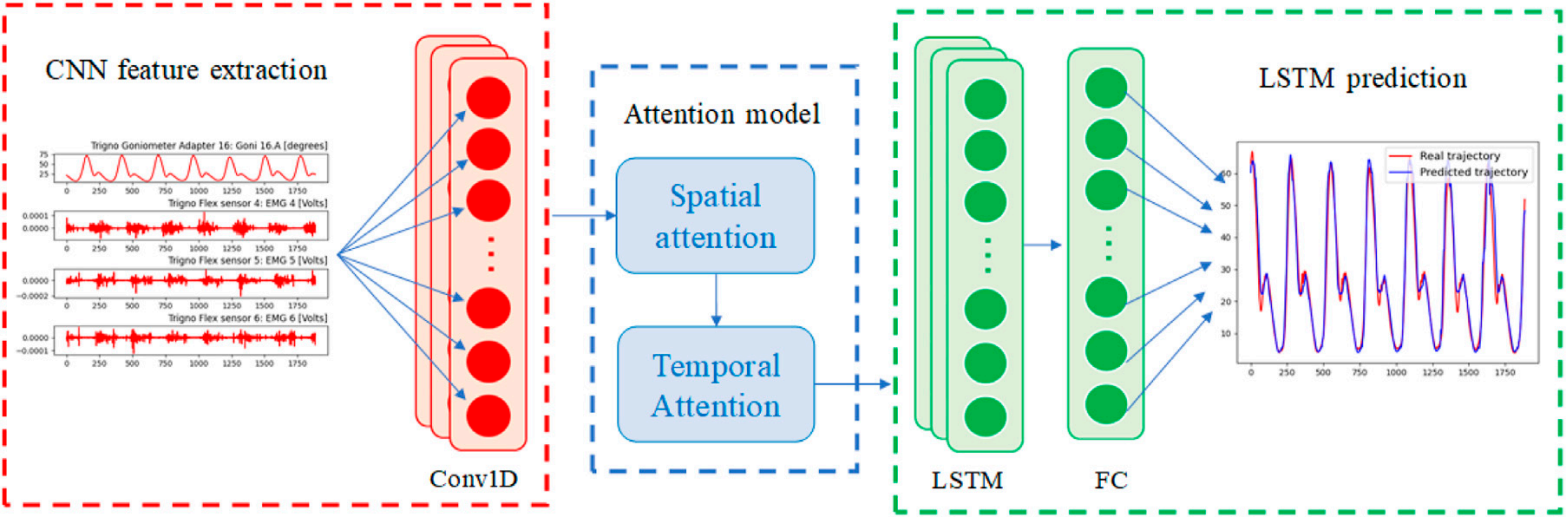
Structure of attention-based CNN-LSTM model.

**FIGURE 5:**
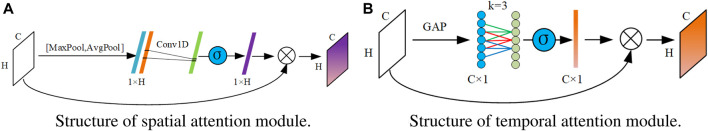
Structure of attention module. **(A)** Structure of spatial attention module. **(B)** Structure of temporal attention module.

The structure of attention-based CNN-LSTM model mainly includes four modules, namely, CNN module, spatial attention module, temporal attention module, LSTM module, as shown in Figure 4. The spatial attention module is as shown in Figure 5A, which refers to the Convolutional Block Attention Module (CBAM) (Woo et al., 2018). The input features are respectively subjected to maximum pooling and average pooling to obtain pooled features $F_{avg}^{s}\in R^{1\times H}$ and $F_{max}^{s}\in R^{1\times H}$ . *H* represent the number of features. Then, the features pass through a 1D convolutional layer with a filter size of 7, and performs a sigmoid function operation to generate a spatial attention weight vector. The attention weight is multiplied element-by-element with the original feature to output the feature vector **M**_**s**_ ∈ *R* ^1×*H*^, as shown in Eq. 1.

$$\begin{array}{cc} M_{s}\left(F\right) & =\sigma \left(f^{7}\left(\left[AvgPool\left(F\right);MaxPool\left(F\right)\right]\right)\right)\\ & =\sigma \left(f^{7}\left(\left[F_{avg}^{s};F_{max}^{s}\right]\right)\right) \end{array}$$ (1)

where *f* ^7^ indicating that the filter size of the convolutional layer is 7, $F_{avg}^{s}$ , $F_{max}^{s}$ represent pooled features after maximum pooling and average pooling operation, respectively.

$$k=\psi \left(C\right)={\left|\frac{\log_{2}\left(C\right)}{\gamma }+\frac{b}{\gamma }\right|}_{\mathrm{odd}}$$ (2)

where the size of the kernel *k* describes the size of the temporal neighborhood, and *γ*, *b* are constants.

The temporal attention module is as shown in Figure 5B, which refers to Squeeze-and-Excitation Network (SENet) (Wang et al., 2020). Firstly, global average pooling is performed on the input features **F** ∈ *R* ^*C*×*H*^, and the dimensions of the input features are mapped from *C* × *H* to *C* × 1. *C* represents the number of time steps. Then, 1D convolution is performed on the features, and the sigmoid function operation is performed to generate a temporal attention weight vector. The attention weight is multiplied element-wise with the original feature to output the feature vector **M**_*t*_ ∈ *R* ^*C*×1^. *k* is adaptive to the number of time steps *C*, determined by Eq. 2, where *γ* = 2, *b* = 1. Longer time steps mean longer distance interactions through mapping *ψ*(*C*).

## 4 Adaptive compliant control strategy

### 4.1 PATH planning

The coordinated gait trajectory of the affected lower limb can be generated based on the information of patient’s healthy lower limb, and the data is collected and inputted into the pre-trained synergetic gait prediction model in actual rehabilitation training. However, it is also necessary to consider the safety problems caused by excessive human-robot interaction force on the affected lower limb. The impedance control can modify the expected trajectory of the exoskeleton through the deviation between expected and actual human-robot interaction force, which can realize that the exoskeleton can move under the guidance of the coordinated gait trajectory as much as possible, while maintaining compliance and reducing the risk of injury.

**FIGURE 6:**
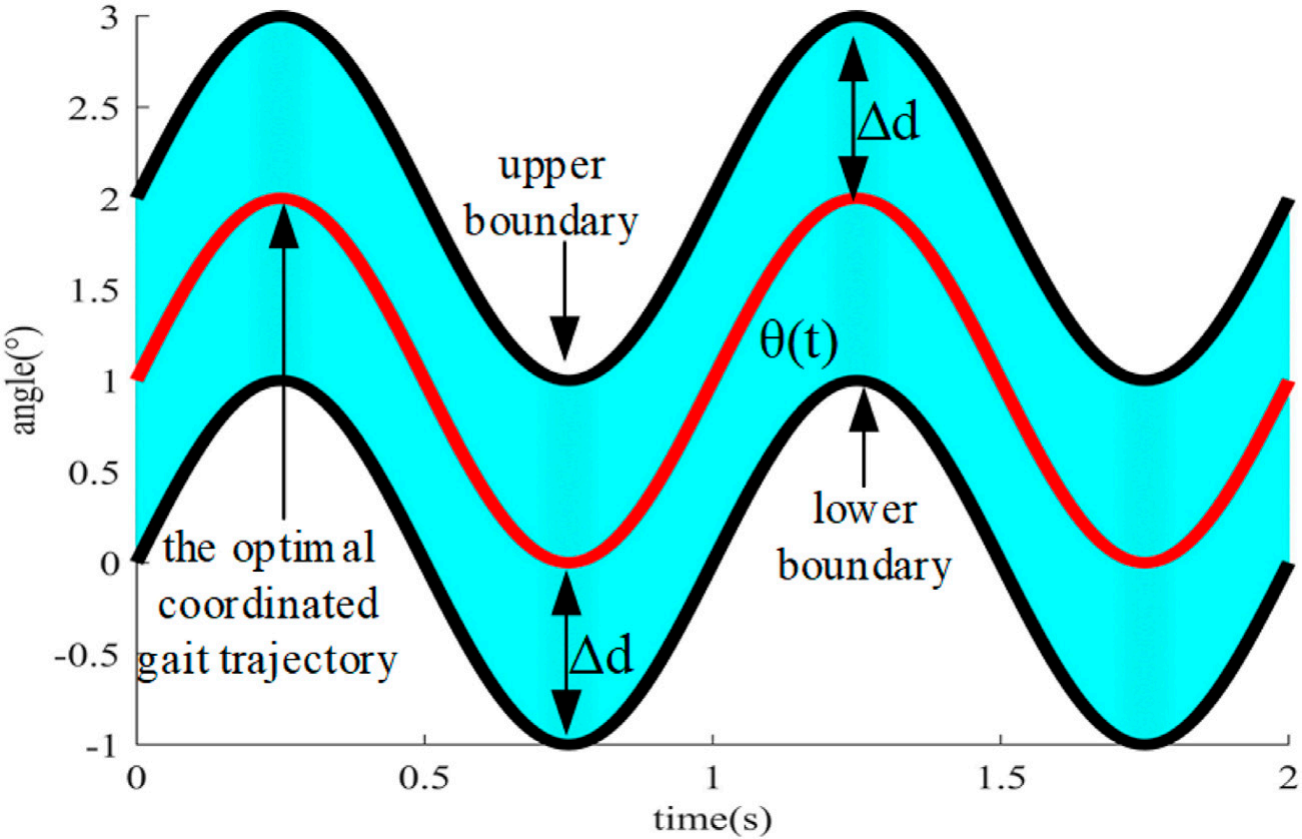
Diagram of coordination space.

As shown in Figure 6, the optimal coordinated gait trajectory is defined as the individualized gait of the affected lower limb generated through synergetic gait prediction model in the specific task or scene. Coordination space is defined as the space that extends outwards with the optimal coordinated gait trajectory as the center, and the movements in the coordination space are all in accordance with normal gait pattern. The robot shows the strong compliance near the optimal coordinated trajectory, and the patient’s motion intention can correct the expected trajectory. When deviating from the optimal coordinated trajectory, the compliance of the robot gradually decreases, but it still follows the optimal coordinated trajectory and ensures that it always cannot exceed the boundary of the coordination space. Meanwhile, the stiffness coefficient needs to be increased. The trajectory is closer to the boundary of coordination space, the faster the impedance parameter increases, and the inertia coefficient and damping coefficient also need to be increased synchronously to ensure the stability of system.

The APF method is widely adopted in obstacle avoidance in path planning, which can make the robot bypass the obstacle and gradually approach the target by controlling the gravitational field and the repulsive field. Similarly, the optimal coordinated gait trajectory is defined as the target, which is generated by synergetic gait prediction model, and the upper and lower boundaries of coordinated space are defined as the obstacles. The gravitational force near the target increases, and the repulsive force near the obstacle increases. The resultant force at the current position serves as the impedance control parameter, enabling small position corrections near the optimal coordinated gait trajectory and extensive position corrections near the boundary of coordination space.

Define the potential function *U*(*p*) of an object at the point *p*, which is the sum of the gravitational potential function *U*_1_(*p*) and the repulsive potential function *U*_2_(*p*), as shown in Eq. 3.

$$U\left(p\right)=U_{1}\left(p\right)+U_{2}\left(p\right)$$ (3)

$$U_{1}\left(p\right)=\frac{1}{2}\varsigma \rho^{2}\left(p,p_{t\arg et}\right)$$ (4)

where *ς* is the gravitational gain factor, and *ρ*(*p*, *p*_*obstacle*_) represents the Euclidean distance between the object and the target.

$$U_{2}\left(p\right)=\frac{1}{2}\eta {\left(\frac{1}{\rho \left(p,p_{\mathrm{obstacle}}\right)}-\frac{1}{\rho_{0}}\right)}^{2}$$ (5)

where *η* is the repulsion gain factor. *ρ*(*p*, *p*_*obstacle*_) represents the Euclidean distance between the object and the obstacle. *ρ*_0_ represents the maximum distance of the repulsion field generated by the obstacle, and 0 ≤ *ρ*(*p*, *p*_*obstacle*_) ≤ *ρ*_0_. When *ρ*(*p*, *p*_*obstacle*_) > *ρ*_0_, *U*_2_(*p*) = 0, and the repulsion field does not work.

When the object is close to the target, the potential function is small and changes slowly, otherwise the potential function is large and changes quickly. When approaching the boundary, the repulsive potential function approaches infinity, which prevents the object from crossing the boundary of coordination space. The potential safety concerns can be raised by infinite impedance. The compliant control gradually increases the impedance as the knee joint approaches the boundary and stops increasing the impedance once the safety threshold has been reached. Therefore, the adaptive impedance control based on the APF is designed as Eq. 6.

$$\left\{\begin{array}{c} M_{d}=M_{d0}+\omega_{1}U\left(t\right)\\ B_{d}=B_{d0}+\omega_{2}U\left(t\right)\\ K_{d}=K_{d0}+\omega_{3}U\left(t\right) \end{array}\right.$$ (6)

where *M*_*d*0_, *B*_*d*0_, and *K*_*d*0_ represent the initial values set by inertia coefficient *M*_*d*_, damping coefficient *B*_*d*_, and stiffness coefficient *K*_*d*_, respectively. *U*(*t*) represents the potential function at the time *t*. *ω*_1_, *ω*_2_ and *ω*_3_ represent the positive weights on the potential function.

### 4.2 Compliant control

The paper employs a single-input single-output model-free adaptive controller (SISO-MFAC) as the position controller to achieve trajectory tracking. SISO-MFAC only adopts the input and output of the controlled system to automatically modify the control signal, which can overcome uncertainty interference and obtain strong robustness against disturbances and unknown model dynamics, as shown in Eq. 7.

$$\left\{\begin{array}{cc} & u\left(k\right)=u\left(k-1\right)+\frac{\rho \phi_{C}\left(k\right)\left(\theta_{d}\left(k+1\right)-\theta \left(k\right)\right)}{\lambda +{\phi_{C}}^{2}\left(k\right)}\\ & {\overset{⌢}{\phi }}_{C}\left(k\right)={\overset{⌢}{\phi }}_{C}\left(k-1\right)\\ & \;\;+\frac{\eta \left(\Delta \theta \left(k\right)-{{\overset{⌢}{\phi }}_{C}}^{T}\left(k-1\right)\Delta u\left(k-1\right)\right)\Delta u\left(k-1\right)}{\mu +{\left|\Delta u\left(k-1\right)\right|}^{2}}\\ & {\overset{⌢}{\phi }}_{C}\left(k\right)={\overset{⌢}{\phi }}_{C}\left(1\right),if\left|{\overset{⌢}{\phi }}_{C}\left(k\right)\right|<bor\left|\Delta u\left(k-1\right)\right|<\text{b }\\ & \;\;\;\;\;orsign\left({\overset{⌢}{\phi }}_{C}\left(k\right)\right)\neq sign\left({\overset{⌢}{\phi }}_{C}\left(1\right)\right) \end{array}\right.$$ (7)

where $u\left(k\right)$ and $\theta \left(k\right)$ represent the input and output of the system at time *k*, respectively. $\phi_{C}\left(k\right)\in R^{m}$ is the pseudo-gradient of the system. $\phi_{C}^{⌢}\left(k\right)$ is an estimate of $\phi_{C}\left(k\right)$ *λ* and *μ* are weighting factors. *ρ* and *η* are step factors. $\phi_{C}^{⌢}\left(1\right)$ is the initial value of $\phi_{C}^{⌢}\left(k\right)$ .

$$\left\{\begin{array}{c} 0<1-\frac{\eta \Delta u^{2}\left(k-1\right)}{\mu +{\left|\Delta u\left(k-1\right)\right|}^{2}}\leq d_{1}<1\\ 0<1-\frac{\rho \phi_{C}\left(k\right){\overset{⌢}{\phi }}_{C}\left(k\right)}{\lambda +{{\overset{⌢}{\phi }}_{C}}^{2}\left(k\right)}\leq d_{2}<1 \end{array}\right.$$ (8)

where *d*_1_ and *d*_2_ are constants.

**FIGURE 7:**
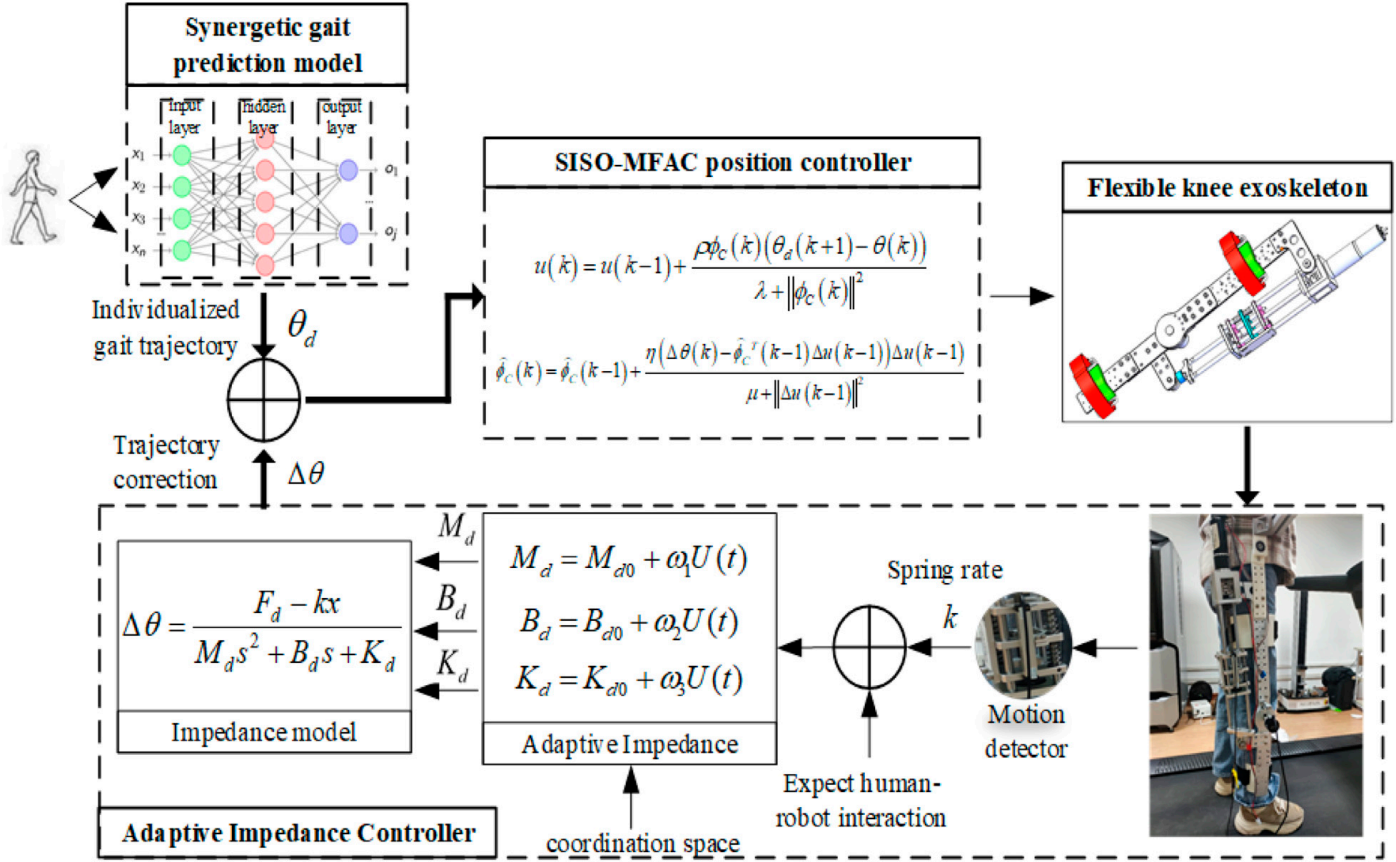
Diagram of the proposed compliant controller.

The diagram of the proposed compliant controller is shown in Figure 7. The compliant controller of knee exoskeleton consists of synergetic gait prediction model, adaptive impedance controller, position controller, and knee exoskeleton. The synergetic gait prediction model is used to generate individualized gait trajectories, and the coordinated gait trajectory of the affected lower limb is generated according to the knee joint angle and sEMG signals of the healthy lower limb. The adaptive impedance controller corrects the expected trajectory in the coordination space according to the deviation between the expected and actual human-robot interaction force. The SISO-MFAC controller can realize the actual trajectory of the exoskeleton to accurately track the expected trajectory. The knee exoskeleton driven by SEA is used as the control object to assist the patient to perform rehabilitation training. Furthermore, the human-robot interaction force between the patient and the exoskeleton is measured by the spring compression at the end of SEA, and the displacement sensor with the range of 50 mm is installed on the spring, which avoids inaccurate measurement due to the relative displacement between the sensor and the human body or the robot. When the actual human-robot interaction force is not equal to the expected human-robot interaction force, the compliant controller generates the correction of expected trajectory, and the position controller controls the exoskeleton to move according to the corrected trajectory. The APF method can ensure that the gait trajectory does not exceed the boundary of coordination space, and the actual trajectory can be guaranteed to be located in the coordination space.

## 5 Experiments and results

### 5.1 Individualized gait trajectory prediction

The sampling frequency of the signal acquisition system is 200 Hz, and the time step is 5 ms, and each sample is 50 s. The knee joint data of the affected lower limb at the current moment is influenced by the healthy lower limb at the previous moment, and there is the delay in actual application from gait prediction to data transmission. After comprehensive considerations, the time step is set to 10, and the knee joint angle of the affected lower limb is predicted based on the information of the healthy lower limb in the previous 100 ms. The dimension of dataset under each task is 8980 × 4. The dataset is split into training (60%), validation (20%) and test (20%) subsets. The input dimension of each dataset is 20 × 4 and the output dimension is 1 × 1. The model is trained based on intra-subjects, and the data is obtained from the trained subject with varying speeds and inclines.

**FIGURE 8:**
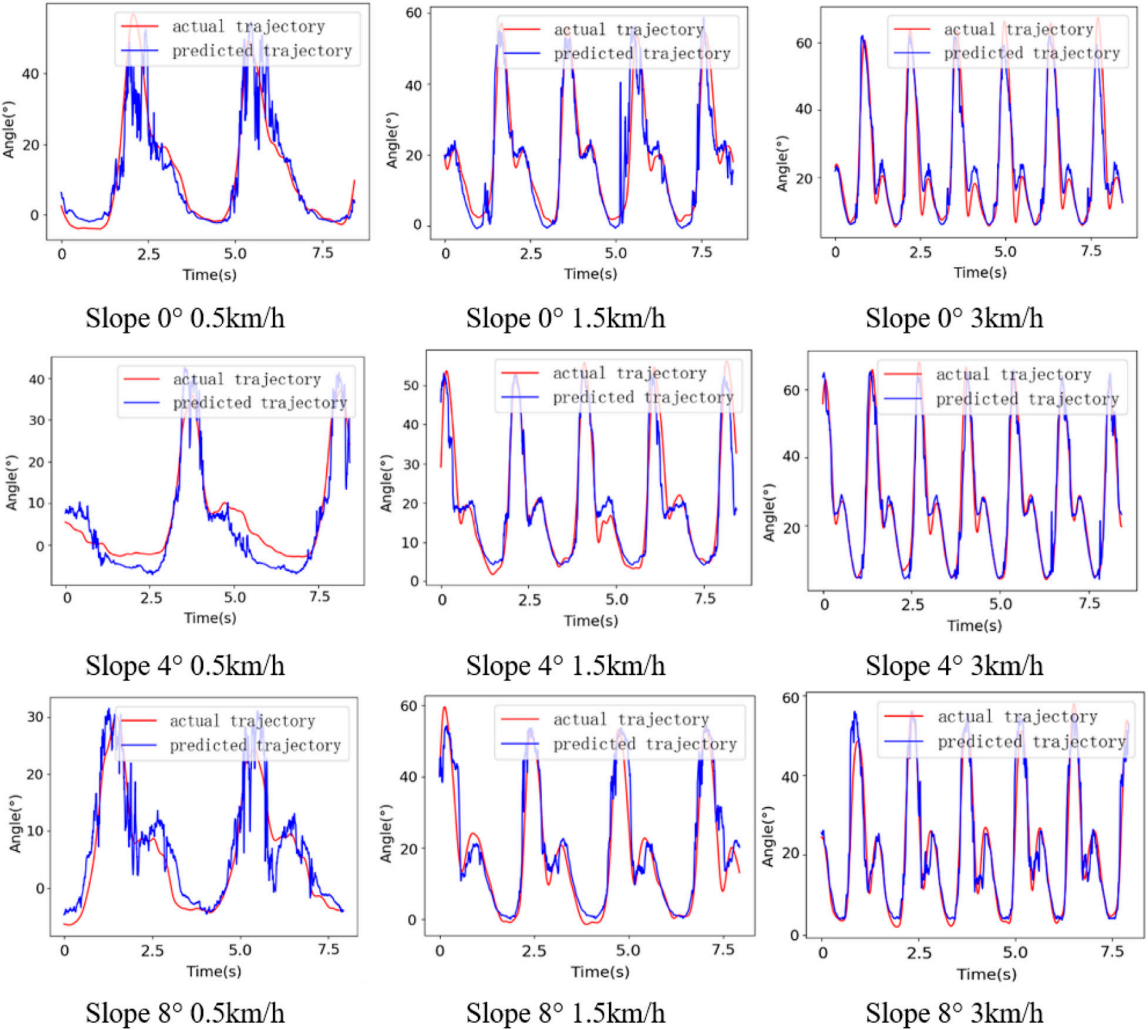
Gait prediction results of subject S1.

Figure 8 shows gait prediction performance of the subject S1 when walking at different speeds on different slopes, in which the red curve represents actual trajectory, and the blue curve represents predicted trajectory. The prediction error is lowest when the speed is 3 km/h, and the prediction performance is worst at the speed of 0.5 km/h. The prediction error at the speed of 0.5 km/h is 42.79% higher than that at the speed of 3 km/h and 12.67% higher than that at the speed of 1.5 km/h. The muscle activity of the subjects is low when walking at low speed, and the periodicity and amplitude of EMG signals is weaker, so the performance of gait prediction at the speed of 0.5 km/h is worst. Similarly, the prediction performance is best when the slope is 8°, and the prediction error is highest on the slope of 0°. Moreover, although the prediction performance is worst at low speed and flat slope, the trend of angle can still be reflected.

**FIGURE 9:**
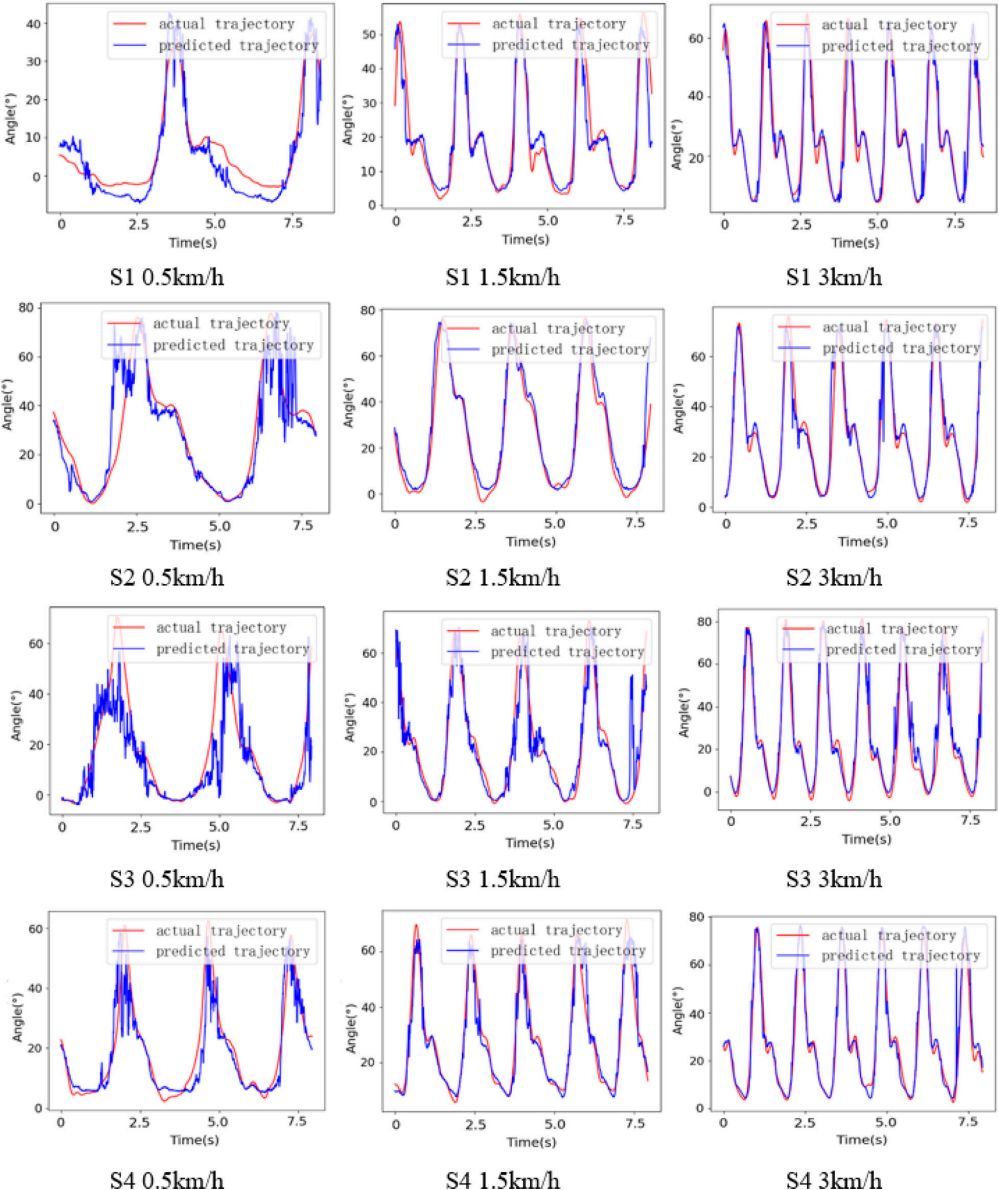
Gait prediction results of different subjects.

To test the applicability of the synergetic gait prediction model, the performance of gait prediction of four subjects at different speeds is analyzed, as shown in Figure 9. The prediction error of S3 is 123.65% higher than that of S1, 64.89% higher than that of S2, and 109.27% higher than that of S4. We have selected five metrics in the time and frequency domains to analyze the sEMG signal, including root mean square (RMS), mean absolute value (MAV), median frequency (MF), mean power frequency (MPF), and signal-to-noise ratio (SNR), and the RMS, MAV, MF, MPF, and SNR metrics show positive correlation with the prediction performance. However, the above metrics are not a dependable basis of prediction performance and can only be adopted as the preliminary reference.

To further quantitatively evaluate the performance, this paper adopts the mean absolute error (MAE) and Pearson correlation coefficient (CC) as the metric. Net1 represents the CNN-LSTM network without attention mechanism, and Net2 refers to the CNN-LSTM network with attention mechanism used in paper (Zhu et al., 2021), and Net is the proposed networks in this paper. The attention mechanism in the Net2 model is the weighted average sum of the output vectors of the LSTM layer. Taking the scene with a slope of 4° as an example, the prediction performance using different model are shown in Table 1. To evaluate the performance of model adopting multi-sensors fusion, the results are shown in Table 2. The Net model adopts the information of the knee joint angle and EMG signal in healthy lower limb as the network input, while the Net3 model only employs the information of the knee joint angle in healthy lower limb.

**TABLE 1:** Performance of gait trajectory prediction using different networks (slope 4°).

Subject	Speed	MAE (°)				CC(%)			
		0.5	1.5	3	Mean	0.5	1.5	3	Mean
S1	Net1	4.081	3.479	2.910	3.490	**96.64**	94.66	97.08	96.13
	Net2	3.685	3.364	2.757	3.269	96.10	**95.96**	97.26	96.44
	CNN	4.019	3.624	3.253	3.632	92.36	95.18	95.21	94.25
	LSTM	4.617	3.973	3.408	3.999	91.01	94.44	96.37	93.94
	Net	**3.574**	**3.172**	**2.503**	**3.083**	96.22	95.64	**97.80**	**96.55**
S2	Net1	7.327	4.749	4.178	5.418	86.75	**97.07**	95.53	93.12
	Net2	6.403	5.403	3.679	5.162	88.18	96.97	96.67	93.94
	CNN	8.434	5.448	4.344	6.075	80.22	96.37	95.53	90.71
	LSTM	9.194	6.005	4.124	6.441	77.39	95.16	95.94	89.49
	Net	**5.855**	**4.429**	**3.395**	**4.600**	**90.44**	96.92	**96.90**	**94.75**
S3	Net1	10.766	9.400	6.006	8.724	67.94	76.65	93.51	79.37
	Net2	10.054	8.070	6.794	8.306	**75.42**	81.52	93.18	83.37
	CNN	12.001	11.462	6.549	10.004	65.76	79.01	92.49	79.08
	LSTM	12.840	12.485	6.933	10.752	60.80	64.34	92.03	72.39
	Net	**9.508**	**7.838**	**5.598**	**7.648**	73.78	**82.52**	**94.34**	**83.55**
S4	Net1	4.272	3.673	2.854	3.600	90.00	**96.54**	98.20	94.91
	Net2	4.080	3.571	3.158	3.603	91.66	96.18	98.24	95.36
	CNN	5.960	4.608	3.438	4.668	85.62	96.44	97.25	93.10
	LSTM	4.916	4.040	3.452	4.136	88.07	95.83	97.17	93.69
	Net	**3.823**	**3.489**	**2.675**	**3.329**	**92.30**	95.74	**98.46**	**95.50**

The bold values represent the optimal performance of the model for each subject on the metric.

**TABLE 2:** Performance of gait trajectory prediction using different input (slope 4°).

Subject	Speed	MAE (°)				CC(%)			
		0.5	1.5	3	Mean	0.5	1.5	3	Mean
S1	Net3	3.861	3.932	3.349	3.714	95.72	92.72	96.08	94.84
	Net	**3.574**	**3.172**	**2.503**	**3.083**	**96.22**	**95.64**	**97.80**	**96.55**
S2	Net3	8.911	6.518	5.976	7.135	77.18	91.48	92.02	86.89
	Net	**5.855**	**4.429**	**3.395**	**4.560**	**90.44**	**96.92**	**96.90**	**94.75**
S3	Net3	10.710	9.183	**5.347**	8.413	69.83	78.41	**95.70**	81.31
	Net	**9.508**	**7.838**	5.598	**7.648**	**73.78**	**82.52**	94.34	**83.55**
S4	Net3	5.138	4.823	3.248	4.403	85.12	90.82	97.34	91.09
	Net	**3.823**	**3.489**	**2.675**	**3.329**	**92.30**	**95.74**	**98.46**	**95.50**

The bold values represent the optimal performance of the model for each subject on the metric.

As shown in Table 1, taking the subject S1 as an example, compared with Net1, the MAE of the model proposed in this paper decrease by 12.42%, 8.82% and 13.99% at 0.5 km/h, 1.5 km/h and 3 km/h, and the CC increase by −0.42% and 0.99%, and 0.72%. Compared with Net2, the MAE of the model proposed in this paper decrease by 3.01%, 5.71% and 9.21%, and the CC increase by 0.12%, −0.32% and 0.18%. The above trend is also reflected in the prediction results of subjects S2, S3 and S4. In summary, the prediction performance of proposed model in this paper is better than Net1 and Net2.

**FIGURE 10:**
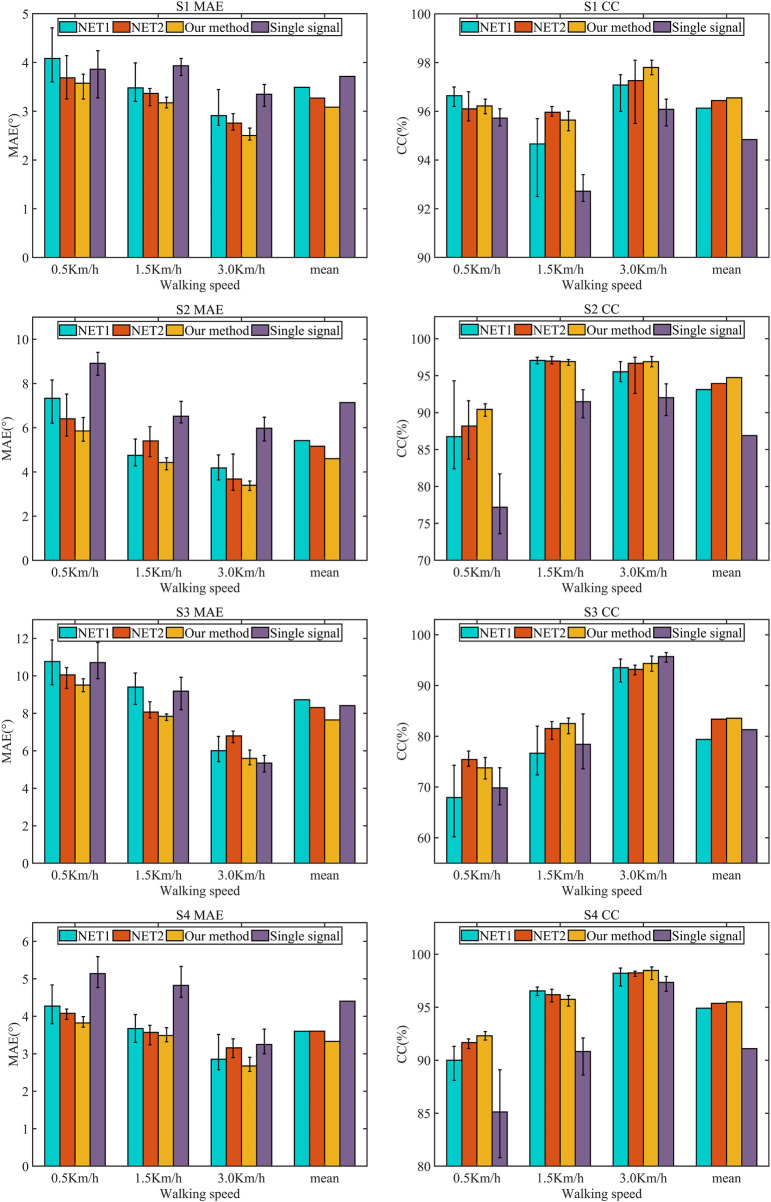
Results of different methods for different subjects at different speeds (slope 4°).

As shown in Table 2, taking the subject S1 as an example, compared with Net3, the MAE of the model adopting multi-sensors input decrease by 7.43%, 19.33%, and 25.26% at 0.5 km/h, 1.5 km/h, and 3 km/h, and CC increase by 0.5%, 2.92% and 1.72%. It is worth noting that although the CC of the model using single input is higher, and the MAE is lower when the subject S3 is at 3 km/h, the mean of CC and MAE at three speeds are still better than Net3. Furthermore, the results of the subjects S1, S2, and S4 are consistent, which shows that multi-sensor fusion can further improve the accuracy of gait prediction. Figure 10 visually shows the prediction results of different methods for different subjects at different speeds on slope 4°. The model using multi-sensors information has smaller MAE and higher CC compared with the model adopting single input. Meanwhile, the mean of MAE at three speeds is smaller than that of the other network, and the mean of CC is higher than that of the other network, which shows that the model proposed in this paper has smaller prediction error and better applicability to different individuals.

### 5.2 Adaptive compliant control

Experiments are carried out on a SEA-driven knee exoskeleton to assess the effectiveness of the proposed compliant control method. Considering the site conditions and safety factors, the experimental scenes are divided into two types, namely, walking on the slope 0° and 4° at the speed of 0.5 km/h, respectively. The boundary range of the coordination space is set to a constant value Δ*d* = 5°, and the distance between the upper and lower boundaries of the coordination space is 10°. To guarantee participant safety, the knee exoskeleton’s motion angle has been limited to −5°–65° degrees *via* the software.

**FIGURE 11:**
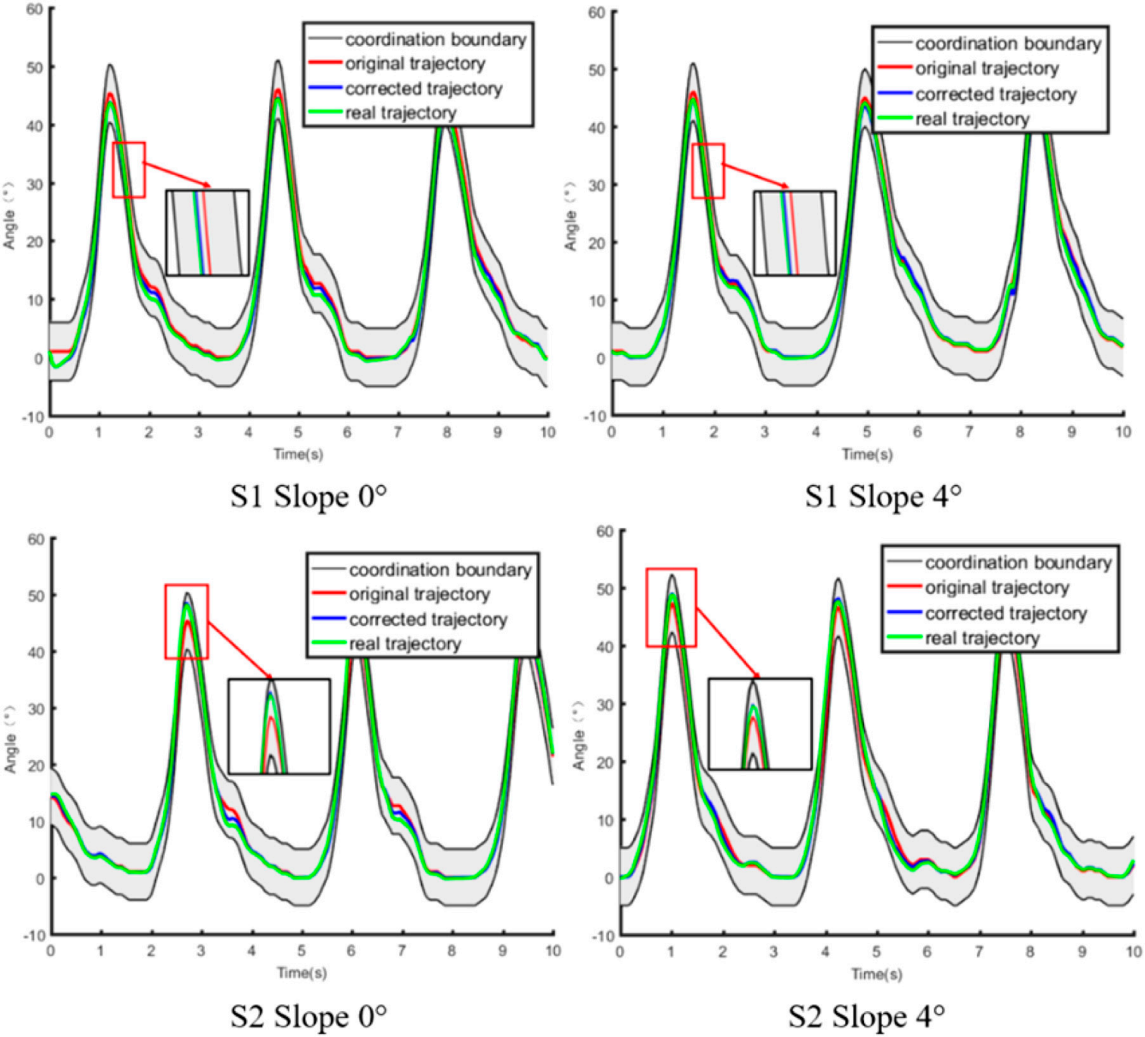
Results of compliant control.

The results of compliant control of the subjects S1 and S2 in different scenes are shown in Figure 11, in which the red curve represents the optimal coordinated trajectory, and the blue curve represents the corrected trajectory, and the green curve represents the actual trajectory of the exoskeleton. The original trajectory is defined as the gait trajectory generated by the prediction model and filtered to comply with the normal human gait pattern. The corrected trajectory represents the trajectory modified by the compliant controller when the actual human-robot interaction force is not equal to the expected human-robot interaction force. The actual trajectory is defined as the angle signal collected by the angle sensor on the knee exoskeleton. Furthermore, the optimal coordinated gait trajectory, the corrected trajectory, and the actual trajectory are all in the coordination space, which proves that the compliant control method can adaptively and nonlinearly modify the impedance parameters according to the actual conditions, and ensure the safety and coordination of rehabilitation training.

**FIGURE 12:**
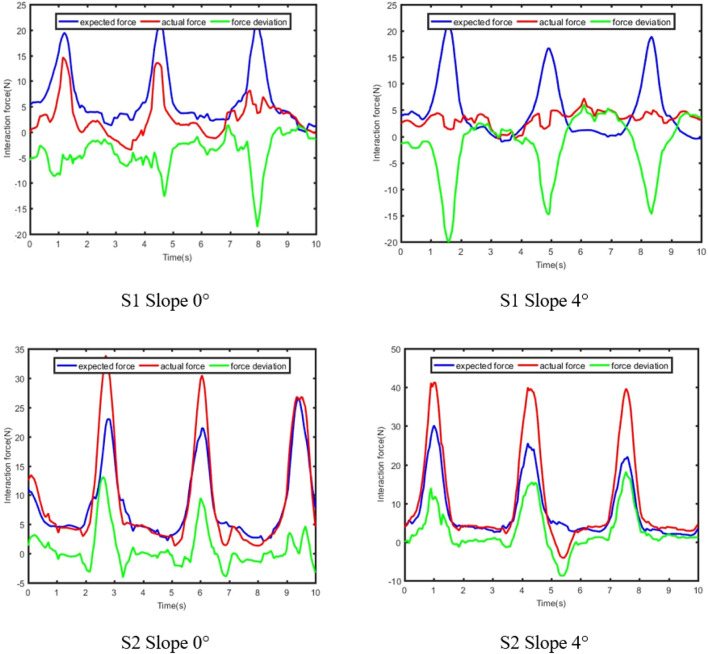
Results of human-robot interaction force.

The results of the human-robot interaction force is shown in Figure 12, in which the blue curve represents the expected human-robot interaction force, and the red curve represents the actual human-robot interaction force, and the green curve represents the deviation between the expected and actual human-robot interaction force. The expected force is defined as the human-robot interaction force of healthy subjects collected by the force sensor in advance. The actual force is defined as the human-robot interaction force of patients during rehabilitation training. Combining Figures 11, 12, it is evident that the trajectory correction is not proportional to the force deviation. The deviation of force is positive, which indicates that the motion intention of the subject is consistent with the direction of the robot.

**FIGURE 13:**
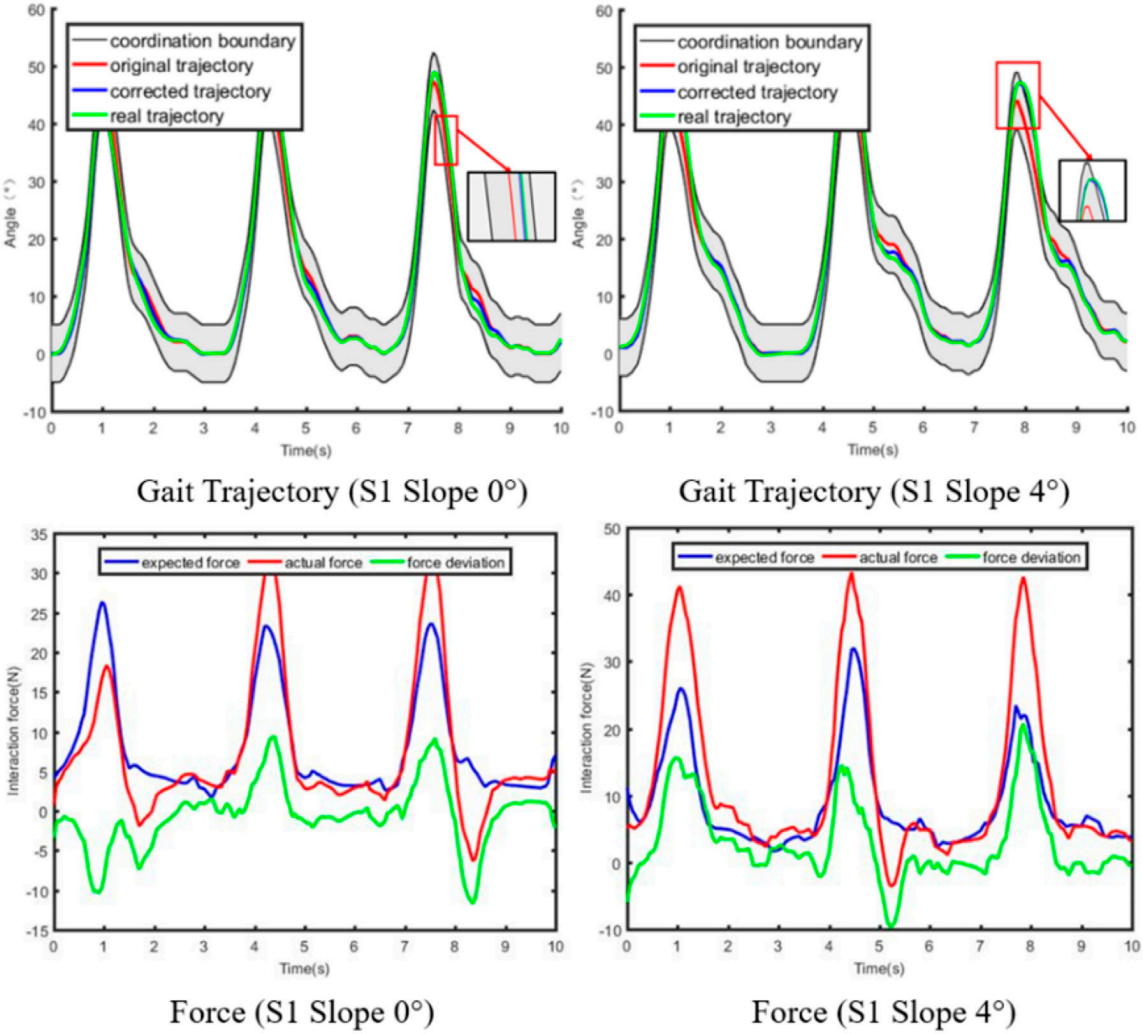
Results of Compliant control with fixed impedance parameters.

The experiment with fixed impedance parameters on the subject S1 are conducted, and the experimental results are shown in Figure 13. When the actual human-robot interaction force deviates from the expected human-robot interaction force, the impedance controller with fixed parameters can also correct the trajectory. However, if the deviation of force is large, the corrected trajectory may exceed the boundary of the coordination space. Consequently, the impedance control system with fixed impedance parameters cannot completely guarantee the safety and coordination of rehabilitation training. The adaptive impedance control proposed in this paper can not only nonlinearly and adaptively modify the expected trajectory according to the human-robot interaction force, but also restrict the actual trajectory to always be in the coordination space.

## 6 Discussion

As an emerging rehabilitation equipment, exoskeleton can reduce the burden on physicians while ensuring the efficacy of rehabilitation, which has gradually become a research hotspot in the field of rehabilitation. However, the current trajectories for rehabilitation are mostly predefined trajectories, which cannot adapt to speed and slope changes during rehabilitation training in the middle and late stages of rehabilitation. Based on the attention-based CNN-LSTM model, the individualized coordinated trajectory of the affected lower limb under different tasks is obtained. The performance of synergetic gait prediction model of different individuals varied widely. Attention mechanism has been proven to effectively improve the performance of the neural network model. Spatial attention and temporal attention modules can assign attention weights to important features. The combined attention mechanism is introduced into synergetic gait prediction model to further improve the prediction accuracy of individualized gait.

Compliant controller is designed based on the guidance of synergetic gait prediction model. The predicted individualized gait trajectory is inputted into the compliant controller as the expected trajectory. The compliant controller can adaptively and nonlinearly modify the impedance parameters according to the distance from the boundary. The correction of trajectory is larger near the expected trajectory, which shows strong compliance. The APF method can ensure that the actual trajectory does not cross the boundary of coordination space while realizing compliant control. There is no clear evidence that compliant control is superior to pure torque/force control or pure position control for stroke rehabilitation. However, patient-dominated training can enhance rehabilitation outcomes, and compliant control can improve patient participation while ensuring patient safety. In addition, the proposed compliant control is mainly used in the middle and late stages of rehabilitation when the patient obtains some motor abilities. Position control is used to assist patients with repetitive passive training, which is mainly used in the early stages of rehabilitation. The advantage of compliant control compared to pure torque/force control lies in the personalized assistance, including the generation of the personalized trajectory and the adaptive modification of impedance parameters in response to actual conditions (Shi et al., 2022; Cao et al., 2024).

The knee exoskeleton driven by SEA designed in this paper has the function of limit protection and size adjustment, which improves the safety and applicability of the equipment. However, the knee exoskeleton is not lightweight, and it is a little cumbersome and inconvenient for patients to use. We aim to decrease the weight and volume of the knee exoskeleton through optimizing the power transmission mode, selecting lightweight high-strength materials such as carbon fiber as the main materials of knee exoskeleton, and combining 3D printing technology. In addition, the application of inter-limb coordination in knee joint rehabilitation is controversial (Vallery et al., 2009; Liang et al., 2018), because the abnormalities in knee joint may alter the features of other joints, ultimately resulting in the deficient desired trajectory of knee joint. Our proposed method can customize the rehabilitation according to the patient’s range of motion and body parameters. Now, we have been actively collaborating with Tongji Hospital, Wuhan, China, to identify eligible patients for the experimental study. Meanwhile, we plan to consider more sensors for multi-level and multi-spatial information complementarity to improve the prediction performance of the subjects with substandard signal quality.

## 7 Conclusion

In this paper, a synergetic gait prediction model based on attention-based CNN-LSTM network and compliant control method based on APF method are proposed. The experimental results show that the proposed synergetic gait prediction model can generate the coordinated gait trajectory, which can achieve lower MAE and higher CC. The subjects move in the coordination space but never cross the coordination boundary. Coordination, compliance, and safety are simultaneously considered in the rehabilitation. In the future, we will design knee exoskeletons that are more lightweight and patient-friendly, test individualized gait prediction model under more scenes, and further verify the effectiveness of the proposed method on patients.

## Data Availability

The original contributions presented in the study are included in the article/Supplementary Material, further inquiries can be directed to the corresponding author.

## References

[B1] Almaghout K. Tarvirdizadeh B. Alipour K. Hadi A. (2022). Rbf neural network-based admittance pd control for knee rehabilitation robot. Robotica 40, 4512–4534. 10.1017/S0263574722001084

[B2] Arami A. Poulakakis-Daktylidis A. Tai Y. F. Burdet E. (2019). Prediction of gait freezing in parkinsonian patients: a binary classification augmented with time series prediction. IEEE Trans. Neural Syst. Rehabilitation Eng. 27, 1909–1919. 10.1109/TNSRE.2019.2933626 31398122

[B3] Asl H. J. Yamashita M. Narikiyo T. Kawanishi M. (2020). Field-based assist-as-needed control schemes for rehabilitation robots. IEEE Trans. Mechatronics 25, 2100–2111. 10.1109/tmech.2020.2992090

[B4] Ballester B. R. Ward N. S. Brander F. Maier M. Kelly K. Verschure P. (2022). Relationship between intensity and recovery in post-stroke rehabilitation: a retrospective analysis. J. Neurology Neurosurg. Psychiatry 93, 226–228. 10.1136/jnnp-2021-326948 PMC8784991 34168083

[B5] Banala S. K. Kim S. H. Agrawal S. K. Scholz J. P. (2009). Robot assisted gait training with active leg exoskeleton (alex). IEEE Trans. Neural Syst. Rehabilitation Eng. 17, 2–8. 10.1109/tnsre.2008.2008280 19211317

[B6] Cao W. Shang D. Yin M. Li X. Xu T. Zhang L. (2023). Development and evaluation of a hip exoskeleton for lateral resistance walk exercise. IEEE Trans. Mechatronics 28, 1966–1974. 10.1109/TMECH.2023.3273717

[B7] Cao Y. Chen X. Zhang M. Huang J. (2024). Adaptive position constrained assist-as-needed control for rehabilitation robots. IEEE Trans. Industrial Electron. 71, 4059–4068. 10.1109/tie.2023.3273270

[B8] Challa S. K. Kumar A. Semwal V. B. Dua N. (2022). An optimized-lstm and rgb-d sensor-based human gait trajectory generator for bipedal robot walking. IEEE Sensors J. 22, 24352–24363. 10.1109/jsen.2022.3222412

[B9] Chen B. Zhong C. H. Zhao X. Ma H. Guan X. Li X. (2017). A wearable exoskeleton suit for motion assistance to paralysed patients. J. Orthop. Transl. 11, 7–18. 10.1016/j.jot.2017.02.007 PMC5866401 29662765

[B10] Chen B. Zi B. Wang Z. Qin L. Liao W.-H. (2019). Knee exoskeletons for gait rehabilitation and human performance augmentation: a state-of-the-art. Mech. Mach. Theory 134, 499–511. 10.1016/j.mechmachtheory.2019.01.016

[B11] Chen L. Wang C. Song X. Wang J. Zhang T. Li X. (2020). Dynamic trajectory adjustment of lower limb exoskeleton in swing phase based on impedance control strategy. Proc. Institution Mech. Eng. Part I J. Syst. Control Eng. 234, 1120–1132. 10.1177/0959651820932026

[B12] Dong Y. Ai Q. Liu H. Meng W. Cheng W. (2022). “Design and control of a sea driven knee exoskeleton for walking assistance,” in 2022 IEEE/ASME International Conference on Advanced Intelligent Mechatronics (AIM), Hokkaido, Japan, July 11th- 15th, 2022, 1243. 10.1109/aim52237.2022.9863380

[B13] Feigin V. L. Brainin M. Norrving B. Martins S. Sacco R. L. Hacke W. (2022). World stroke organization (wso): global stroke fact sheet 2022. Int. J. Stroke 17, 18–29. 10.1177/17474930211065917 34986727

[B14] Gautam A. Panwar M. Biswas D. Acharyya A. (2020). Myonet: a transfer-learning-based lrcn for lower limb movement recognition and knee joint angle prediction for remote monitoring of rehabilitation progress from semg. IEEE J. Transl. Eng. Health Med. 8, 1–10. 10.1109/JTEHM.2020.2972523 PMC7062147 32190428

[B15] Huang P. Li Z. Zhou M. Li X. Cheng M. (2022). Fuzzy enhanced adaptive admittance control of a wearable walking exoskeleton with step trajectory shaping. IEEE Trans. Fuzzy Syst. 30, 1541–1552. 10.1109/tfuzz.2022.3162700

[B16] Kim J. Lee G. Heimgartner R. Revi D. A. Karavas N. Nathanson D. (2019). Reducing the metabolic rate of walking and running with a versatile, portable exosuit. Sci. Robotics 365, 668–672. 10.1126/science.aav7536 31416958

[B17] Kim J. Moon J. H. Kim J. (2021). Impedance control of human ankle joint with electrically stimulated antagonistic muscle co-contraction. IEEE Trans. Neural Syst. Rehabilitation Eng. 29, 1593–1603. 10.1109/TNSRE.2021.3104091 34379593

[B18] Kong K. Bae J. Tomizuka M. (2012). A compact rotary series elastic actuator for human assistive systems. IEEE Trans. Mechatronics 17, 288–297. 10.1109/tmech.2010.2100046

[B19] Li X. Liu Y.-H. Yu H. (2018). Iterative learning impedance control for rehabilitation robots driven by series elastic actuators. Automatica 90, 1–7. 10.1016/j.automatica.2017.12.031

[B20] Liang F.-Y. Zhong C.-H. Zhao X. Castro D. L. Chen B. Gao F. (2018). “Online adaptive and lstm-based trajectory generation of lower limb exoskeletons for stroke rehabilitation,” in IEEE International Conference on Robotics and Biomimetics, Kuala Lumpur, Malaysia, December 12-15, 2018, 27–32. 10.1109/ROBIO.2018.8664778

[B21] Liu D.-X. Wu X. Du W. Wang C. Chen C. Xu T. (2017). Deep spatial-temporal model for rehabilitation gait: optimal trajectory generation for knee joint of lower-limb exoskeleton. Assem. Autom. 37, 369–378. 10.1108/aa-11-2016-155

[B22] Liu J. Wang C. He B. Li P. Wu X. (2022). Metric learning for robust gait phase recognition for a lower limb exoskeleton robot based on semg. IEEE Trans. Med. Robotics Bionics 4, 472–479. 10.1109/tmrb.2022.3166543

[B23] Liu L. Leonhardt S. Ngo C. Misgeld B. J. E. (2020). Impedance-controlled variable stiffness actuator for lower limb robot applications. IEEE Trans. Automation Sci. Eng. 17, 991–1004. 10.1109/tase.2019.2954769

[B24] Mazumder O. Kundu A. S. Lenka P. K. Bhaumik S. (2016). Multi-channel fusion based adaptive gait trajectory generation using wearable sensors. J. Intelligent Robotic Syst. 86, 335–351. 10.1007/s10846-016-0436-y

[B25] Morbidoni C. Cucchiarelli A. Agostini V. Knaflitz M. Fioretti S. Di Nardo F. (2021). Machine-learning-based prediction of gait events from emg in cerebral palsy children. IEEE Trans. Neural Syst. Rehabilitation Eng. 29, 819–830. 10.1109/TNSRE.2021.3076366 33909568

[B26] Mounir Boudali A. Sinclair P. J. Manchester I. R. (2019). Predicting transitioning walking gaits: hip and knee joint trajectories from the motion of walking canes. IEEE Trans. Neural Syst. Rehabilitation Eng. 27, 1791–1800. 10.1109/TNSRE.2019.2933896 31398125

[B27] Na J. Kim H. Lee G. Nam W. (2023). Deep domain adaptation, pseudo-labeling, and shallow network for accurate and fast gait prediction of unlabeled datasets. IEEE Trans. Neural Syst. Rehabilitation Eng. 31, 2448–2456. 10.1109/TNSRE.2023.3272887 37141069

[B28] Perez-Ibarra J. C. Siqueira A. A. G. Silva-Couto M. A. de Russo T. L. Krebs H. I. (2019). Adaptive impedance control applied to robot-aided neuro-rehabilitation of the ankle. IEEE Robotics Automation Lett. 4, 185–192. 10.1109/lra.2018.2885165

[B29] Schumacher M. Wojtusch J. Beckerle P. von Stryk O. (2019). An introductory review of active compliant control. Robotics Aut. Syst. 119, 185–200. 10.1016/j.robot.2019.06.009

[B30] Shi D. Li L. Zhang W. Ding X. (2022). Field-based human-centred control on so(3) for assist-as-needed robotic rehabilitation. IEEE Trans. Med. Robotics Bionics 4, 785–795. 10.1109/tmrb.2022.3194372

[B31] Sivakumar S. Gopalai A. A. Lim K. H. Gouwanda D. (2019). Artificial neural network based ankle joint angle estimation using instrumented foot insoles. Biomed. Signal Process. Control 54, 101614. 10.1016/j.bspc.2019.101614

[B32] Song J. Zhu A. Tu Y. Zhang X. Cao G. (2023a). Novel design and control of a crank-slider series elastic actuated knee exoskeleton for compliant human–robot interaction. IEEE Trans. Mechatronics 28, 531–542. 10.1109/tmech.2022.3204921

[B33] Song W. Zhao P. Li X. Deng X. Zi B. (2023b). Data-driven design of a six-bar lower-limb rehabilitation mechanism based on gait trajectory prediction. IEEE Trans. Neural Syst. Rehabilitation Eng. 31, 109–118. 10.1109/TNSRE.2022.3217448 36288218

[B34] Spyrakos-Papastavridis E. Dai J. S. (2021). Minimally model-based trajectory tracking and variable impedance control of flexible-joint robots. IEEE Trans. Industrial Electron. 68, 6031–6041. 10.1109/tie.2020.2994886

[B35] Sridar S. Qiao Z. Muthukrishnan N. Zhang W. Polygerinos P. (2018). A soft-inflatable exosuit for knee rehabilitation: assisting swing phase during walking. Front. Robotics AI 5, 44. 10.3389/frobt.2018.00044 PMC7805964 33500930

[B36] Sun T. Peng L. Cheng L. Hou Z. G. Pan Y. (2020). Composite learning enhanced robot impedance control. IEEE Trans. Neural Netw. Learn. Syst. 31, 1052–1059. 10.1109/TNNLS.2019.2912212 31107667

[B37] Tanghe K. De Groote F. Lefeber D. De Schutter J. Aertbelien E. (2020). Gait trajectory and event prediction from state estimation for exoskeletons during gait. IEEE Trans. Neural Syst. Rehabilitation Eng. 28, 211–220. 10.1109/TNSRE.2019.2950309 31675336

[B38] Thakur D. Biswas S. (2022). Attention-based deep learning framework for hemiplegic gait prediction with smartphone sensors. IEEE Sensors J. 22, 11979–11988. 10.1109/jsen.2022.3172603

[B39] Vallery H. van Asseldonk E. H. Buss M. van der Kooij H. (2009). Reference trajectory generation for rehabilitation robots: complementary limb motion estimation. IEEE Trans. Neural Syst. Rehabilitation Eng. 17, 23–30. 10.1109/TNSRE.2008.2008278 19211320

[B40] Wang K. Y. Ma F. C. Hao M. Zhang L. X. Liu P. (2011). Experimental research on force/position control of a wire-driven parallel rehabilitative robot. Appl. Mech. Mater. 138-139, 68–73. 10.4028/www.scientific.net/AMM.138-139.68

[B41] Wang Q. Wu B. Zhu P. Li P. Zuo W. Hu Q. (2020). “Eca-net: efficient channel attention for deep convolutional neural networks,” in 2020 IEEE/CVF Conference on Computer Vision and Pattern Recognition (CVPR), Seattle, WA, USA, June 13 2020 to June 19 2020. 10.1109/cvpr42600.2020.01155

[B42] Wang Y. Yang Y. Zhao B. Qi X. Hu Y. Li B. (2021). Variable admittance control based on trajectory prediction of human hand motion for physical human-robot interaction. Appl. Sci. 11, 5651. 10.3390/app11125651

[B43] Wang Z. Zhu M. Su Z. Guan B. Wang A. Wang Y. (2017). Post-stroke depression: different characteristics based on follow-up stage and gender-a cohort perspective study from mainland China. Neurological Res. 39, 996–1005. 10.1080/01616412.2017.1364514 28828931

[B44] Woo S. Park J. Lee J.-Y. Kweon I. S. (2018). “Cbam: convolutional block attention module,” in European conference on computer vision (ECCV), Munich, Germany, September 8-14, 2018. 10.1007/978-3-030-01234-2_1

[B45] Xiong D. Zhang D. Zhao X. Zhao Y. (2021). Deep learning for emg-based human-machine interaction: a review. IEEE/CAA J. Automatica Sinica 8, 512–533. 10.1109/jas.2021.1003865

[B46] Xu Y. Yang W. Chen M. Chen S. Huang L. (2022). Attention-based gait recognition and walking direction estimation in wi-fi networks. IEEE Trans. Mob. Comput. 21, 465–479. 10.1109/tmc.2020.3012784

[B47] Yan Y. Liu G. Zhang L. Gong R. Fu P. Han B. (2022). Biomechanical effect of valgus knee braces on the treatment of medial gonarthrosis: a systematic review. Appl. Bionics Biomechanics 2022, 1–15. 10.1155/2022/4194472 PMC9168205 35677195

[B48] Yang L. Xiang K. Pang M. Yin M. Wu X. Cao W. (2023). Inertial sensing for lateral walking gait detection and application in lateral resistance exoskeleton. IEEE Trans. Instrum. Meas. 72, 1–14. 10.1109/TIM.2023.3265105 37323850

[B49] Yang Q. Xie C. Tang R. Liu H. Song R. (2020). Hybrid active control with human intention detection of an upper-limb cable-driven rehabilitation robot. IEEE Access 8, 195206–195215. 10.1109/access.2020.3033301

[B50] Yu Y.-L. Lan C.-C. (2019). Design of a miniature series elastic actuator for bilateral teleoperations requiring accurate torque sensing and control. IEEE Robotics Automation Lett. 4, 500–507. 10.1109/lra.2019.2891287

[B51] Zhang C. Huang M. Z. Kehs G. J. Braun R. G. Cole J. W. Zhang L. Q. (2021). Intensive in-bed sensorimotor rehabilitation of early subacute stroke survivors with severe hemiplegia using a wearable robot. IEEE Trans. Neural Syst. Rehabilitation Eng. 29, 2252–2259. 10.1109/TNSRE.2021.3121204 PMC8843010 34665733

[B52] Zhu C. Liu Q. Meng W. Ai Q. Xie S. (2021). “An attentionbased cnn-lstm model with limb synergy for joint angles prediction,” in 2021 IEEE/ASME International Conference on Advanced Intelligent Mechatronics (AIM), Delft, Netherlands, 12-16 July 2021, 747–752. 10.1109/aim46487.2021.9517544

[B53] Zhu C. Luo L. Mai J. Wang Q. (2022). Recognizing continuous multiple degrees of freedom foot movements with inertial sensors. IEEE Trans. Neural Syst. Rehabilitation Eng. 30, 431–440. 10.1109/TNSRE.2022.3149793 35130162

[B54] Zou C. Huang R. Peng Z. Qiu J. Cheng H. (2021). “Synergetic gait prediction for stroke rehabilitation with varying walking speeds,” in 2021 IEEE/RSJ International Conference on Intelligent Robots and Systems (IROS), Prague, Czech Republic, September 27 - Oct. 1, 2021, 7231. 10.1109/iros51168.2021.9635860

