# Supplementary material for: Synergetic gait prediction and compliant control of SEA-driven knee exoskeleton for gait rehabilitation
Source: Front Bioeng Biotechnol. 2024 Jan 26;12:1358022. doi: 10.3389/fbioe.2024.1358022 (PMC10853324; doi:10.3389/fbioe.2024.1358022)
Supplement: Supplementary file 1 [file DataSheet1.PDF]

Supplementary Material

1 SUPPLEMENTARY DATA

Supplementary Material should be uploaded separately on submission. Please include any supplementary data, figures and/or tables. All supplementary files are deposited to FigShare for permanent storage and receive a DOI.

Supplementary material is not typeset so please ensure that all information is clearly presented, the appropriate caption is included in the file and not in the manuscript, and that the style conforms to the rest of the article. To avoid discrepancies between the published article and the supplementary material, please do not add the title, author list, affiliations or correspondence in the supplementary files.

2 SUPPLEMENTARY TABLES AND FIGURES

For more information on Supplementary Material and for details on the different file types accepted, please see the Supplementary Material section of the Author Guidelines.

Figures, tables, and images will be published under a Creative Commons CC-BY licence and permission must be obtained for use of copyrighted material from other sources (including re-published/adapted/modified/partial figures and images from the internet). It is the responsibility of the authors to acquire the licenses, to follow any citation instructions requested by third-party rights holders, and cover any supplementary charges.

2.1 Figures

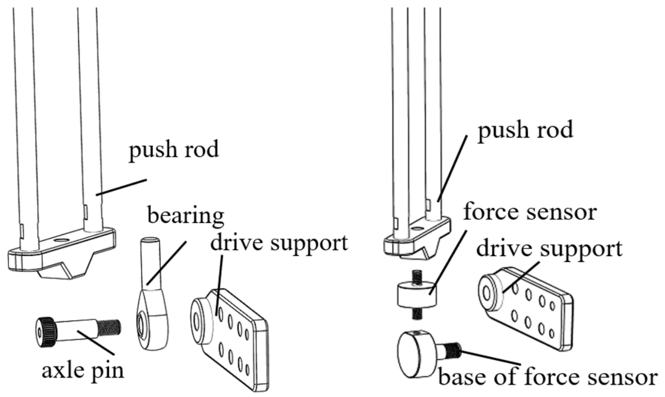

Figure S1. The dual-purpose interface.

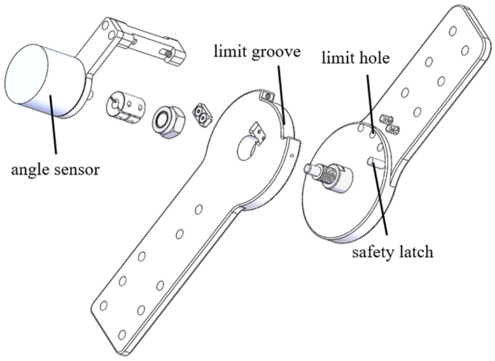

Figure S2. The safety mechanism.

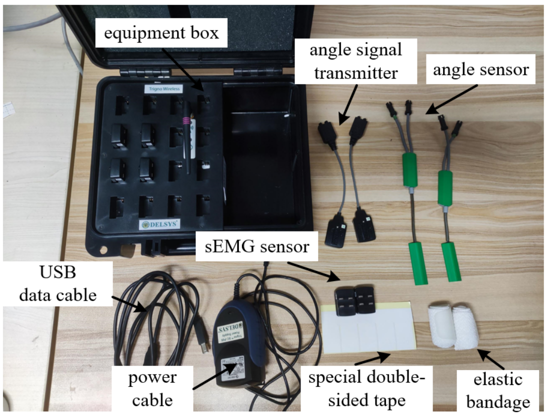

Figure S3. sEMG signal acquisition system.

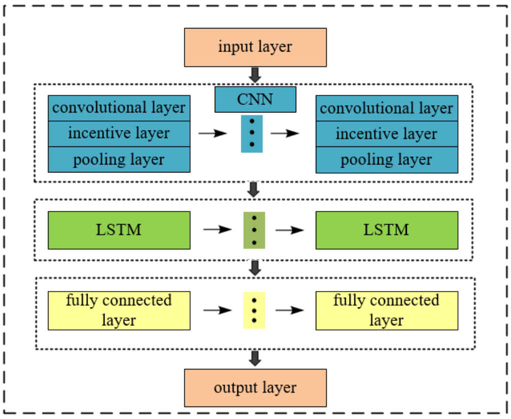

Figure S4. Structure of hybrid CNN-LSTM model.

Table S1. Information of Subjects

Subject	Gender	Age	Height(cm)	Weight(kg)	Leg length(cm)
S1	male	25	166	65	92
S2	male	25	173	63	98
S3	male	25	178	75	101
S4	male	23	164	65	90

Table S2. Time-domain and frequency-domain indicators of EMG signals of four subjects

	S1			S2			S3			S4		
	BF	VL	RF	BF	VL	RF	BF	VL	RF	BF	VL	RF
RMS	$1.393*10^{-5}$	$2.115*10^{-5}$	$1.260*10^{-5}$	$2.688*10^{-5}$	$1.258*10^{-5}$	$8.474*10^{-6}$	$1.931*10^{-5}$	$1.139*10^{-5}$	$3.875*10^{-6}$	$6.316*10^{-6}$	$5.885*10^{-6}$	$3.928*10^{-6}$
MAV	$8.047*10^{-6}$	$1.092*10^{-5}$	$6.732*10^{-6}$	$1.625*10^{-5}$	$6.578*10^{-6}$	$5.336*10^{-6}$	$9.201*10^{-6}$	$5.864*10^{-6}$	$2.342*10^{-6}$	$3.131*10^{-6}$	$3.088*10^{-6}$	$2.302*10^{-6}$
ZC	0.068	0.078	0.040	0.048	0.057	0.021	0.064	0.069	0.024	0.037	0.045	0.017
MF	111.500	115.900	40.500	29.500	83.100	23.300	121.900	131.900	100.500	82.900	36.900	41.300
MPF	172.925	60.858	55.251	124.254	20.212	74.090	149.719	68.782	47.742	106.508	19.806	18.761
RMSE	$1.200*10^{-5}$	$5.800*10^{-5}$	$2.700*10^{-5}$	$4.700*10^{-5}$	$5.700*10^{-5}$	$3.500*10^{-5}$	$1.400*10^{-5}$	$5.900*10^{-5}$	$2.900*10^{-5}$	$1.200*10^{-5}$	$5.600*10^{-5}$	$2.700*10^{-5}$
SNR	1.616	-8.742	-6.500	-4.870	-13.188	-12.384	2.888	-14.112	-16.703	-5.205	-19.634	-16.781

BF= biceps femoris; VL=vastus lateralis; RF= rectus femoris; RMS= root mean square; MAV = mean absolute value; MF =median frequency; MPF = mean power frequency; SNR = signal-to-noise ratio; ZC = zero crossing; RMSE = root-mean-square error.
